# Functional Recovery From Prolonged Severe Paraparesis in Patients With Spinal Dural Arteriovenous Fistula: A Report of Two Cases

**DOI:** 10.7759/cureus.89727

**Published:** 2025-08-10

**Authors:** Ippei Kitade, Yasutaka Mizukami, Makoto Isozaki, Hidetaka Arishima, Kenichiro Kikuta

**Affiliations:** 1 Division of Rehabilitation Medicine, University of Fukui Hospital, Eiheiji, JPN; 2 Department of Community Medicine, University of Fukui, Eiheiji, JPN; 3 Department of Neurosurgery, University of Fukui, Eiheiji, JPN

**Keywords:** gait disturbance, prolonged disease duration, rehabilitation therapy, severe paraparesis, spinal dural arteriovenous fistula (sdavf)

## Abstract

A spinal dural arteriovenous fistula (sdAVF) is a rare spinal vascular condition often associated with delayed diagnosis and progressive myelopathy. Long disease duration and severe motor deficits before treatment initiation are generally associated with poor functional outcomes. We report two cases of sdAVF in patients presenting with prolonged symptom duration and severe lower limb paralysis. Despite these unfavourable prognostic indicators, both patients underwent early postoperative rehabilitation aimed at activating the central pattern generator through intensive weight-bearing exercises following surgical treatment. In Case 1, where concomitant spinal degenerative disease was not present, the patient achieved rapid functional improvement and independent ambulation within one week. In contrast, in Case 2, which was complicated by spinal stenosis, ossification of the posterior longitudinal ligament, and poor obliteration of the abnormal vasculature, the patient demonstrated delayed but steady functional gains, ultimately achieving ambulation with minimal assistance at 13 months postoperatively. These cases suggest that even in patients with sdAVF presenting with long-standing symptoms and severe paralysis, functional recovery is possible with tailored rehabilitation. Complications such as spinal degenerative disorders and incomplete obliteration of the abnormal vasculature may affect the rate and degree of recovery. Early and sustained rehabilitation is essential for managing such high-risk patients.

## Introduction

Spinal dural arteriovenous fistula (sdAVF) is an acquired condition and a rare form of spinal arteriovenous shunt. An sdAVF is an abnormal connection between a dural artery and vein near the spinal cord, leading to increased venous pressure and spinal cord congestion, which causes progressive neurological deficits [1,2]. If appropriate treatment is not administered, spinal cord function can be irreversibly compromised [2,3].

Treatment options for sdAVF include surgical intervention (interruption of the reflux veins) and embolization. However, the optimal treatment strategy for patients with sdAVF remains controversial [4]. Moreover, the disease is often challenging to diagnose definitively, which can worsen the physical prognosis [1,3,5,6]. A longer duration of symptoms prior to intervention, as well as the presence of severe motor deficits at the time of treatment initiation, are associated with poorer functional outcomes [1,6-8]. Therefore, rehabilitation programs for patients with sdAVF must be tailored and adjusted according to prognosis. However, Iovtchev et al. reported that the potential for functional ambulation was poor despite prolonged rehabilitation treatment in the late diagnosis of sdAVF [9]. Most previous studies on the treatment of sdAVF do not include descriptions of the rehabilitation programs following surgical treatment or embolization. Additionally, information regarding the time elapsed between the treatment of the fistula and initiation of physical therapy and rehabilitation is often lacking [10].

In light of these challenges, we describe two cases of patients with sdAVF who presented with a prolonged disease course and severe lower extremity paralysis. After surgery, one patient achieved a high level of gait function in the early phase, whereas the other did not. Herein, we discuss the rehabilitation (physical therapy) course and outcomes of these patients.

## Case presentation

Case 1

**Figure 1:**
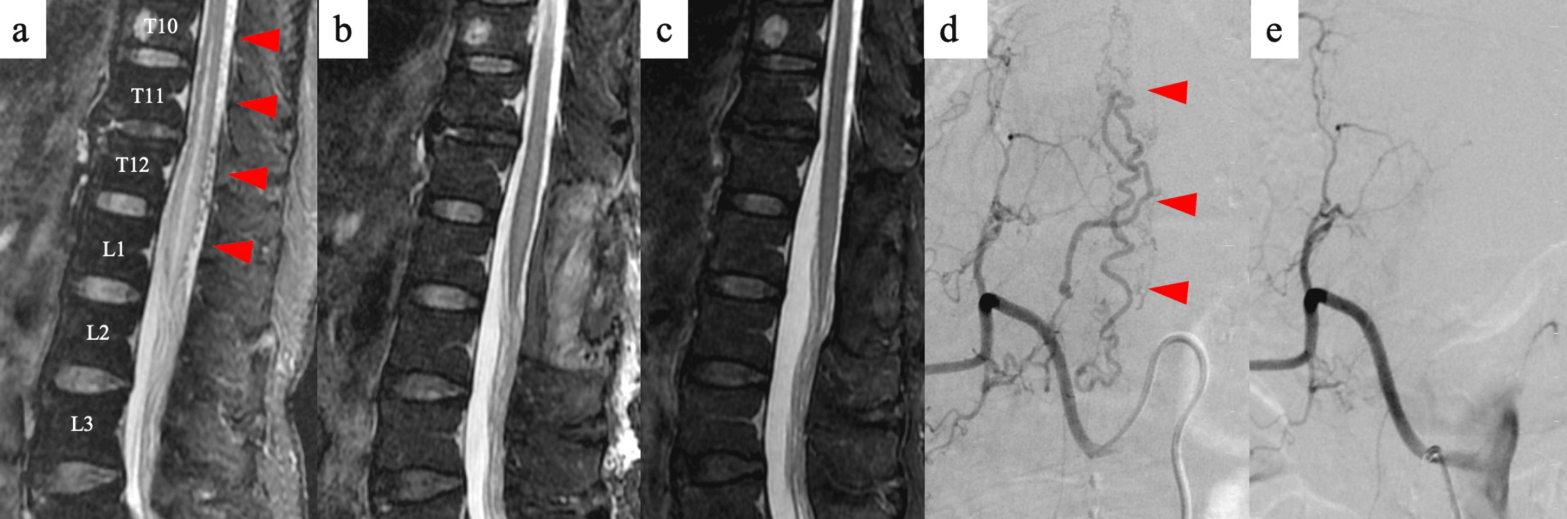
Imaging findings before and after surgery in Case 1 (a) T2-weighted MRI scan before surgery showing a high signal area in the spinal cord and dilated vessel on the dorsal surface of the spinal cord; (b) Postoperative T2-weighted MRI does not show a high signal area in the spinal cord and dilated vessel on the dorsal surface of the spinal cord; (c) T2-weighted MRI scan at three years after surgery, during the follow-up evaluation, does not show a high signal area in the spinal cord and dilated vessel on the dorsal surface of the spinal cord; (d) Angiography before surgery shows an sdAVF with L1-2 right root artery as the feeding vessel; (e) Angiography after surgery does not show an sdAVF with L1-2 right root artery as the feeding vessel. The red arrowheads indicate findings of a spinal dural arteriovenous fistula.

A 64-year-old woman who was experiencing weakness in the bilateral lower extremities for seven months visited her local doctor and was referred to our hospital. On the Aminoff-Logue disability scale (ALS) [11], Gait G5 and numbness/hypesthesia below L4 were observed. She could not stand and walk independently with/without an aid and/or device, was unable to perform activities of daily living (ADL) normally, and spent the entire day in bed with full assistance (Spinal Cord Independence Measure (SCIM) [12]: 8/100 points, ALS-micturition M2, ALS-bowel B1). The ALS and the SCIM were used in this study with permission obtained from the original publishers. The patient's patellar and Achilles tendon reflexes were hyperactive, and a decrease in balance ability was observed (Berg Balance Scale (BBS) [13]: 5/56 points). The patient was using a wheelchair for locomotion. T2-weighted magnetic resonance imaging (MRI) showed a high-signal area in the spinal cord and a dilated vessel on the dorsal surface of the spinal cord (Figure 1a). Angiography revealed an sdAVF with L1-2 right root artery as the feeding vessel (Figure 1d). A diagnosis of sdAVF was made based on these imaging findings (Figures 1a, 1d).

The patient underwent T11/L1 laminectomy, and occlusion of the draining vein and the arteriovenous shunt. T2-weighted MRI after surgery did not reveal a high-signal area in the spinal cord or dilated vessels on the dorsal surface of the spinal cord (Figure 1b). Angiography after surgery revealed an sdAVF with the L1-2 right root artery as the feeding vessel disappeared (Figure 1e). 

The patient wished to undergo postoperative rehabilitation to promote functional recovery. The postoperative rehabilitation program was designed to enhance ADLs by facilitating antigravity training, such as sitting and standing, and alternating lower limb movement, with the aim of activating the central pattern generator [14], which is considered to underlie spinal cord function. Two days after surgery, she started undergoing a rehabilitation program comprising sitting and standing exercises, including muscle strength exercises for both legs. Three days after surgery, a reduction in the lower limb tendon reflex hyperactivity was observed, and the patient could walk using a walker (American Spinal Injury Association Impairment Scale (AIS): D). Her motor and sensory functions at seven days after surgery changed substantially compared with those before surgery (ALS-gait G0, AIS: E “independent ambulation”, BBS: 54/56 points, numbness/hypesthesia below L4, SCIM: 79/100 points). Twenty-four days after surgery, upon discharge, the sensory disturbance in both lower limbs and urinary dysfunction had resolved (ALS-micturition M0, ALS-bowel B0), and the patient was independent in ADL (SCIM: 98/100 points). Rehabilitation treatment was completed at the time of discharge (24 days after surgery).

After discharge, regular follow-ups with the attending neurosurgeon continued. Subsequently, over the three-year period following surgery, physical examination revealed that the patient remained independent in ADL without any deterioration (AIS: E, ALS-gait G0, ALS-micturition M1, ALS-bowel B0, SCIM: 98/100 points). Furthermore, imaging findings continued to show resolution of spinal cord enlargement, abnormal signals, and abnormal blood vessels (Figure 1c). Three years after the surgery, the scheduled follow-up was completed.

Case 2

**Figure 2:**
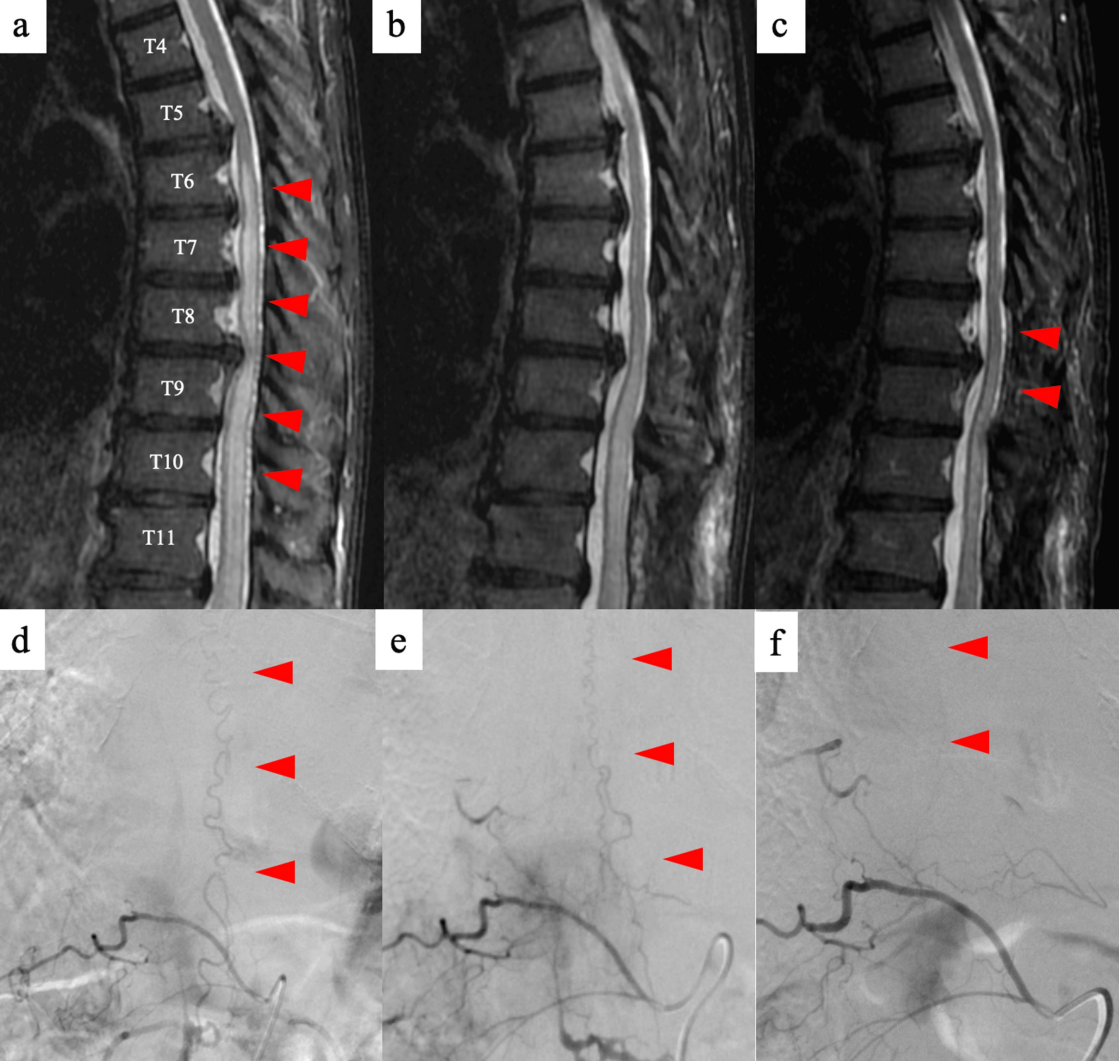
Imaging findings before and after surgery, and after embolization in Case 2 (a) T2-weighted MRI shows hyperintense areas within the spinal cord at and below the T6/7 level, with flow voids noted along the dorsal aspect of the cord from T8 to T11. Spinal cord compression is observed at the T5/6, T6/7, T7/8, and T8/9 intervertebral levels, with particularly severe stenosis noted at the T8/9 level; (b) Postoperative T2-weighted MRI; (c) T2-weighted MRI scan after embolization (nine months after surgery) shows persistent high signal intensity within the spinal cord; (d) Angiography before surgery shows an sdAVF with T11-12 right root artery as the feeding vessel; (e) Angiography after surgery shows a persisting spinal arterio-venous fistula fed by the right radicular artery (dorsal somatic branch) of right T11 intercostal; (f) Angiography after embolization (nine months after surgery) shows a marked reduction in abnormal vasculature, although minimal residual shunting remains. The red arrowheads indicate findings of a spinal dural arteriovenous fistula.

A 68-year-old man had been experiencing weakness in both lower extremities for four months and was admitted for improvement of paraplegia. The patient exhibited weakness in both lower limbs and was unable to perform tasks such as standing up, transferring, maintaining a standing posture, or walking independently (ALS-gait G5, AIS: C, manual muscle testing (MMT) of bilateral lower limbs: 1-2). Sensory disturbances, including touch, pain, proprioception, vibration, deep sensation below T10, and urinary dysfunction were observed (ALS-micturition M2, ALS-bowel B1). The patellar and Achilles tendon reflexes of both lower extremities were absent. T2-weighted MRI revealed hyperintense areas within the spinal cord at and below the T6/7 level, with flow voids along the dorsal aspect of the cord from T8 to T11 (Figure 2a). Spinal cord compression was observed at the T5/6, T6/7, T7/8, and T8/9 intervertebral levels, with severe stenosis at the T8/9 level. Angiography revealed an sdAVF with T11-12 right root artery as the feeding vessel (Figure 2d). Steroids were administered after admission, but no drastic improvement in the symptoms was observed. Additionally, rehabilitation therapy was incorporated, and as a result of the two-week period leading up to the surgery, the patient could practice walking (10 m) with moderate assistance using a walker. However, the practical benefits were limited, and there was no significant improvement. The other symptoms showed no notable improvement.

The patient underwent T8/9 and T11/12 laminectomy and occlusion of the draining vein and the arteriovenous shunt. However, T2-weighted MRI and angiography after surgery revealed a spinal arteriovenous fistula fed by the right radicular artery (dorsal somatic branch) in the right T11 intercostal space (Figures 2b, 2e). The patient wished to undergo postoperative rehabilitation to promote functional recovery. The postoperative rehabilitation program was designed to enhance ADL by facilitating antigravity training, such as sitting and standing, and alternating lower limb movement, with the aim of activating the central pattern generator [14], which is considered to underlie spinal cord function. However, the patient developed a high fever postoperatively, and concerns about possible cerebrospinal fluid leakage led to a delay in rehabilitation.

Rehabilitation therapy was initiated one week after surgery, once the fever had resolved. Starting from one week post surgery, weight-bearing exercises (standing exercises) were primarily focused on improving trunk and lower limb coordination, progressing to gait exercises. By the second week after surgery, the patient acquired alternating lower limb stepping motions and, with minimal assistance, was able to walk for 40 m using a walker (AIS: D). Although there was no change in the level of assistance required by three weeks post surgery, the continuous walking distance increased to 100 m. Four weeks after surgery, the patient was able to walk independently using a walker (50 m). Five weeks after the surgery, the patient was temporarily transferred to a rehabilitation hospital. Following transfer back to our hospital from the rehabilitation hospital 21 weeks after surgery, the patient reached a significant milestone 22 weeks after surgery. At this point, he was able to walk independently for 40 m using a four-point cane in both hands and was fully independent of ambulation using a walker covering a distance of over 350 m. However, ambulation remained functionally limited, and the patient continued to rely on a wheelchair for mobility in daily life. At 22 weeks after surgery, the patient was discharged (ALS-gait G5, ALS-micturition M1, and ALS-bowel B1).

Seven months after the surgery, during the follow-up evaluation, T2-weighted MRI and angiography showed that the spinal arteriovenous fistula was fed by the right radicular artery (dorsal somatic branch) of the right T11 intercostal space. Although there was no deterioration in the patient’s symptoms nine months after surgery, the patient underwent embolization at T11/12. Postoperative MRI revealed persistently high signal intensity within the spinal cord, whereas postoperative angiography showed a marked reduction in the abnormal vasculature, although minimal residual shunting remained (Figures 2c, 2f). The patient was discharged home four days after embolization. Following discharge, the patient received rehabilitation three times per week through outpatient rehabilitation services. Eleven months after surgery (two months after embolization), the patient could walk with a single cane. The patient had significantly reduced wheelchair use and adopted a lifestyle with minimal reliance on wheelchairs 13 months after surgery (four months after embolization) (ALS-gait G4, ALS-micturition M1, and ALS-bowel B0).

## Discussion

sdAVF is a rare condition among spinal arteriovenous shunts, often resulting in delayed diagnosis and has been reported to negatively affect clinical outcomes owing to treatment postponement [3,5,6]. Regarding symptoms, Gogu et al. reported that patients with sdAVF develop progressive myelopathy and experience considerable neurological deficits [2]. Regarding functional outcomes, it is generally believed that a shorter interval between symptom onset and treatment is associated with better post-treatment recovery from paralysis, although there are some opposing views [1,7]. Furthermore, aside from disease duration, preoperative ALS gait scores have also been reported to be associated with postoperative clinical improvement in patients with sdAVF [6,8,15]. Regarding the timing of intervention, Ofran et al. emphasized the importance of early intervention, including rehabilitation, in patients with sdAVF, stating that the potential for functional ambulation is related to the timing of intervention [6]. However, information regarding the time elapsed between fistula treatment and the beginning of physical therapy and rehabilitation is also lacking in most series [15].

Although surgical and endovascular treatments are commonly discussed as first-line therapies for patients with sdAVF [4], the incorporation of postoperative rehabilitation is reported to be essential for maximising functional outcomes [16]. However, reports detailing the specific postoperative rehabilitation courses are limited. In most studies that mention rehabilitation, the specific content of the rehabilitation programs is not clearly outlined [10,17-19]. Sucuoğlu et al. described a specific postoperative rehabilitation protocol, which included passive and active-assisted range of motion exercises, muscle strengthening training, neuromuscular electrical stimulation, and balance and coordination exercises initiated from the first postoperative week [19]. However, the final outcomes have not been reported. Shimizu et al. reported that a three-month rehabilitation program using the Hybrid Assistive Limb® (HAL®) (Cyberdyne Inc., Tsukuba, Ibaraki, Japan) in a patient with sdAVF, starting six months after surgery, led to improvements in gait speed, cadence, and lower limb muscle activity [20]. Although HAL is a specialised and expensive device not applicable to routine rehabilitation, their report demonstrates that intensive rehabilitation, even during the chronic phase, can yield significant functional improvement.

We reported the cases of two patients with sdAVF, both of whom presented with relatively long disease duration and severe motor deficits, as reflected by their high ALS gait scores. In both patients, postoperative rehabilitation involving intensive weight-bearing exercises was initiated during the early postoperative phase. Case 1 showed rapid improvement; the patient achieved independent ambulation within one week after surgery, and by three weeks, both sensory disturbances and urinary dysfunction had resolved, indicating a favourable recovery. In contrast, Case 2 required 13 months postoperatively to regain independence from wheelchair use. Postoperative imaging in Case 1 demonstrated complete resolution of the spinal cord hyperintensity and disappearance of the dilated perimedullary veins. However, in Case 2, spinal cord hyperintensity and perimedullary vascular engorgement persisted immediately after surgery. We considered that the presence of a residual shunt was possible. Additionally, this patient had coexisting ossification of the posterior longitudinal ligament, which may have contributed to the overlapping symptoms. Despite this overlap, we observed gradual functional recovery, including improvements in gait and ADL during the period before reoperation, which was attributed to continued rehabilitation, albeit at a slower pace. Following reoperation, further regression of the abnormal vasculature was observed, accompanied by additional improvement in ambulation and ADL. Furthermore, the two patients with sdAVF in this report presented with symptoms at the thoracolumbar level. However, not only rehabilitation, including proactive weight-bearing exercises aimed at activating the central pattern generator, but also adjustment of the rehabilitation approach based on the level of the spinal lesion may be important. For example, cervical-level lesions may require interventions targeting spasticity, thoracic-level lesions may require treatment for ataxia, and lumbar-level lesions may require rehabilitation focused on muscle weakness or paralysis.

## Conclusions

These findings suggest that, although the pace of recovery may vary among individuals, the addition of rehabilitation therapy involving intensive weight-bearing exercises may have the potential to lead to gradual functional improvement even in patients with sdAVF presenting with long-standing disease and severe paralysis. This study was limited to a case report of two patients with long-standing disease and severe paralysis. Therefore, further studies involving larger patient populations are warranted to determine the most effective treatment strategies.

## References

[1] Van Dijk JM, TerBrugge KG, Willinsky RA, Farb RI, Wallace MC (2002). Multidisciplinary management of spinal dural arteriovenous fistulas: clinical presentation and long-term follow-up in 49 patients. Stroke.

[2] Gogu AE, Pusztai A, Stroe AZ, Docu Axelerad D, Docu Axelerad A (2020). Back pain in rare diseases: a comparison of neck and back pain between spinal cord ischemia and spinal dural arteriovenous fistula. Brain Sci.

[3] Ronald AA, Yao B, Winkelman RD, Piraino D, Masaryk TJ, Krishnaney AA (2020). Spinal dural arteriovenous fistula: diagnosis, outcomes, and prognostic factors. World Neurosurg.

[4] Sasamori T, Hida K, Yano S, Asano T, Seki T, Houkin K (2016). Long-term outcomes after surgical and endovascular treatment of spinal dural arteriovenous fistulae. Eur Spine J.

[5] Klopper HB, Surdell DL, Thorell WE (2009). Type I spinal dural arteriovenous fistulas: historical review and illustrative case. Neurosurg Focus.

[6] Ofran Y, Yovchev I, Hiller N, Cohen J, Rubin SA, Schwartz I, Meiner Z (2013). Correlation between time to diagnosis and rehabilitation outcomes in patients with spinal dural arteriovenous fistula. J Spinal Cord Med.

[7] Song JK, Gobin P, Duckwiler GR, Murayama Y, Frazee JG, Martin NA, Viñuela F (2002). N-butyl 2-cyanoacrylate embolization of spinal dural arteriovenous fistulae. AJNR Am J Neuroradiol.

[8] Peng Y, Ren Y, Hou J (2023). Clinical outcomes and prognostic factors in the surgical treatment of spinal dural arteriovenous fistulas: a retrospective study of 118 patients. Sci Rep.

[9] Iovtchev I, Hiller N, Ofran Y, Schwartz I, Cohen J, Rubin SA, Meiner Z (2015). Late diagnosis of spinal dural arteriovenous fistulas resulting in severe lower-extremity weakness: a case series. Spine J.

[10] Prieto R, Pascual JM, Gutiérrez R, Santos E (2009). Recovery from paraplegia after the treatment of spinal dural arteriovenous fistula: case report and review of the literature. Acta Neurochir (Wien).

[11] Aminoff MJ, Logue V (1974). Clinical features of spinal vascular malformations. Brain.

[12] Catz A, Itzkovich M, Agranov E, Ring H, Tamir A (1997). SCIM--spinal cord independence measure: a new disability scale for patients with spinal cord lesions. Spinal Cord.

[13] Berg K, Wood-Dauphinée S, Williams JI, Gayton D (1989). Measuring balance in the elderly: preliminary development of an instrument. Physuither Can.

[14] Dimitrijevic MR, Gerasimenko Y, Pinter MM (1998). Evidence for a spinal central pattern generator in humans. Ann N Y Acad Sci.

[15] Özkan N, Kreitschmann-Andermahr I, Goerike SL (2015). Single center experience with treatment of spinal dural arteriovenous fistulas. Neurosurg Rev.

[16] Lemke DM, Hacein-Bey L (2004). Atypical spinal cord injury: spinal dural arteriovenous fistula. SCI Nurs.

[17] Sun HS, Yun HS, Song MK, Han JY, Choi IS, Lee SG (2011). Dural arteriovenous fistula on the brain stem and upper cervical spinal cord - a case report -. Ann Rehabil Med.

[18] Gonzalez-Morgado D, de Dios-Lascuevas M, Blasco-Casado F, Segura-Navarro X, Tomasello-Weitz A, Piñana C, Haddad S (2024). Spinal arteriovenous fistula leading to acute paraplegia after a lumbar nerve root block: successful embolization with complete neurological recovery-a case report. Interv Neuroradiol.

[19] Sucuoğlu H, Aktürk A (2020). Spinal dural arteriovenous fistula: a rare cause of progressive myelopathy and bladder and bowel dysfunction. Turk J Phys Med Rehabil.

[20] Shimizu Y, Nakai K, Kadone H (2018). The Hybrid Assistive Limb® intervention for a postoperative patient with spinal dural arteriovenous fistula and chronic spinal cord injury: A case study. J Spinal Cord Med.

